# Homozygous in-frame deletion in *CATSPERE* in a man producing spermatozoa with loss of CatSper function and compromised fertilizing capacity

**DOI:** 10.1093/humrep/dey278

**Published:** 2018-09-17

**Authors:** Sean G Brown, Melissa R Miller, Polina V Lishko, Douglas H Lester, Stephen J Publicover, Christopher L R Barratt, Sarah Martins Da Silva

**Affiliations:** 1 School of Science, Engineering & Technology, Abertay University, Dundee, UK; 2 Department of Molecular and Cell Biology, University of California, Berkeley, CA, USA; 3 School of Biosciences, the University of Birmingham, Birmingham, UK; 4 Reproductive and Developmental Biology, School of Medicine, Ninewells Hospital and Medical School, University of Dundee, Dundee, UK; 5 Assisted Conception Unit, Ninewells Hospital, Dundee, UK

**Keywords:** Calcium signaling, infertility, CatSper, spermatozoa, mutation

## Abstract

**STUDY QUESTION:**

Does a man (patient 1) with a previously described deficiency in principle cation channel of sperm (CatSper) function have a mutation in the CatSper-epsilon (*CATSPERE*) and/or CatSper-zeta (*CATSPERZ*) gene?

**SUMMARY ANSWER:**

Patient 1 has a homozygous in-frame 6-bp deletion in exon 18 (c.2393_2398delCTATGG, rs761237686) of *CATSPERE.*

**WHAT IS KNOWN ALREADY:**

CatSper is the principal calcium channel of mammalian spermatozoa. Spermatozoa from patient 1 had a specific loss of CatSper function and were unable to fertilize at IVF. Loss of CatSper function could not be attributed to genetic abnormalities in coding regions of seven CatSper subunits. Two additional subunits (CatSper-epsilon (*CATPSERE*) and CatSper-zeta (*CATSPERZ*)) were recently identified, and are now proposed to contribute to the formation of the mature channel complex.

**STUDY DESIGN, SIZE, DURATION:**

This was a basic medical research study analysing genomic data from a single patient (patient 1) for defects in *CATSPERE* and *CATSPERZ*.

**PARTICIPANTS/MATERIALS, SETTING, METHODS:**

The original exome sequencing data for patient 1 were analysed for mutations in *CATSPERE* and *CATSPERZ*. Sanger sequencing was conducted to confirm the presence of a rare variant.

**MAIN RESULTS AND THE ROLE OF CHANCE:**

Patient 1 is homozygous for an in-frame 6-bp deletion in exon 18 (c.2393_2398delCTATGG, rs761237686) of *CATSPERE* that is predicted to be highly deleterious.

**LIMITATIONS, REASONS FOR CAUTION:**

The nature of the molecular deficit caused by the rs761237686 variant and whether it is exclusively responsible for the loss of CatSper function remain to be elucidated.

**WIDER IMPLICATIONS OF THE FINDINGS:**

Population genetics are available for a significant number of predicted deleterious variants of CatSper subunits. The consequence of homozygous and compound heterozygous forms on sperm fertilization potential could be significant. Selective targeting of CatSper subunit expression maybe a feasible strategy for the development of novel contraceptives.

**STUDY FUNDING/COMPETING INTEREST(S):**

This study was funded by project grants from the MRC (MR/K013343/1 and MR/012492/1), Chief Scientist Office/NHS research Scotland. This work was also supported by NIH R01GM111802, Pew Biomedical Scholars Award 00028642 and Packer Wentz Endowment Will to P.V.L. C.L.R.B is the editor-in-chief of Molecular Human Reproduction, has received lecturing fees from Merck and Ferring, and is on the Scientific Advisory Panel for Ohana BioSciences. C.L.R.B was chair of the World Health Organization Expert Synthesis Group on Diagnosis of Male infertility (2012–2016).

## Introduction

Human CatSper is a highly complex progesterone-sensitive calcium channel that is expressed in the principle piece of the sperm flagellum (Lishko and Mannowetz, 2018). While evidence from CatSper knock-out mice implicates it as essential for male fertility (Ren *et al.*, 2001; Qi *et al.*, 2007; Chung *et al.*, 2011), attempts to identify equivalent naturally occurring mutations in infertile men have produced equivocal results. Large genomic deletions and compounding issues with spermatogenesis in such patients result in multiple sperm defects making it impossible to conclude that loss of CatSper *per se* was sufficient to cause infertility (Avidan *et al.*, 2003; Smith *et al.*, 2013).

In a previous study we used progesterone-mediated calcium influx as a ‘marker’ of CatSper function to screen for patients with ‘normal’ semen parameters but failure of CatSper function (Williams *et al.*, 2015). We reported that spermatozoa from one man (patient 1) had a stable lesion in CatSper function and failed to fertilize at IVF. Specifically, spermatozoa from patient 1 failed to produce any CatSper-related ion currents and failed to respond with calcium influx when stimulated with progesterone (Williams *et al.*, 2015). Of particular note was that we did not observe any genetic abnormalities that could result in the reported phenotype in any of the coding regions of CatSper subunits.

Recently two new CatSper subunits have been identified: CatSper-epsilon and CatSper zeta. Based on mouse gene knock-out studies, CatSper-zeta has been confirmed to have a role in mouse fertilization competence (Chung *et al.*, 2017). However, the importance of CatSper-epsilon remains to be verified: a lesion in this subunit may correlate with failed CatSper function. We report here that indeed the exome sequence analysis revealed a homozygous in-frame deletion in the putative extracellular coding region of *CATSPER epsilon (CATSPERE)* and hypothesize that it is the cause of loss of CatSper conductance and subfertility in patient 1.

## Materials and Methods

Analysis was conducted on genomic DNA and sequencing data obtained previously (Williams *et al.*, 2015). Patient 1 is of white European ethnicity from non-consanguineous parents.

### Bioinformatics

*Normal CATSPERE genomic Sanger SCF trace* (https://trace.ncbi.nlm.nih.gov/Traces/home/) was compared with a Sanger sequencing standard chromatogram format (SCF) file generated from patient 1 DNA (http://bioedit.software.informer.com/7.2/). CatSper-epsilon evolutionary distant orthologues (http://www.uniprot.org/ and https://www.ncbi.nlm.nih.gov/gene/) were aligned (https://www.ebi.ac.uk/Tools/msa/muscle/). The generated CLUSTAL multiple sequence alignment was imported and edited (http://www.softpedia.com/get/Science-CAD/GeneDoc.shtml). A search for conserved structural domains within CatSper-epsilon (https://www.ncbi.nlm.nih.gov/Structure/cdd/wrpsb.cgi) indicated that residues I699-P902 align well (*E* value = 1.65e-23) with the pfam15020 conserved extracellular CatSper-delta superfamily domain. Therefore, this sequence was aligned with the corresponding CatSper-delta sequence (K515-Q718) to calculate sequence similarity. A pathogenicity score was generated (http://provean.jcvi.org/seq_submit.php).

### Sanger sequencing

To confirm the *CATSPERE* variant in patient 1 genomic DNA, exon 18 was amplified by PCR using ThermoPrime ReddyMix PCR Master Mix (Fisher Scientific, Loughborough, Leicestershire, UK) and the bespoke primers (5′–3′—F:CATCCAGCTGTCAAAAGACAC, R:CTACCCACTGCTGCCTTATTC) under the following conditions; 95°C for 10 min, followed by a program of 94°C for 30 s, 53°C for 30 s and 72°C for 30 s for 35 cycles, and ending with a 10 min extension at 72°C. The expected 431 bp amplicon, was confirmed by gel electrophoresis. The remaining PCR amplicon was purified using a QIAquick PCR Purification Kit (Qiagen, Skelton House, Manchester, UK) and analysed by bidirectional sequencing using the same primers.

## Results

**Figure 1:**
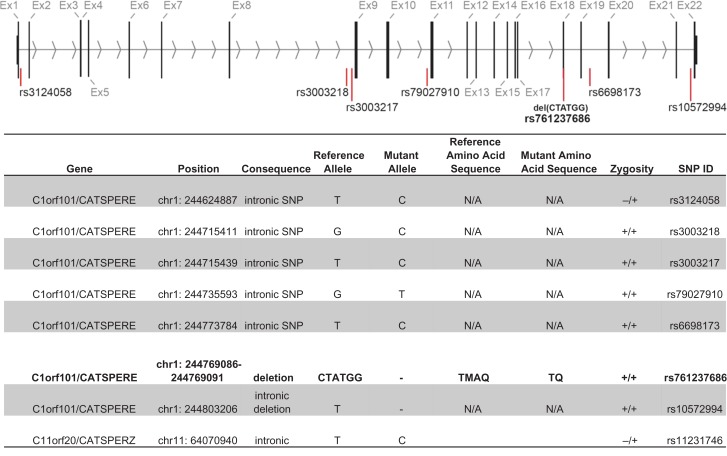
Sequence variation summary information for *CATSPERE* and *CATSPERZ* from patient 1. The exome sequencing identification of a homozygous pathogenic 6 bp deletion (CTATGG, rs761237686) in cation channels sperm associated epsilon (*CATSPERE)* exon 18, of patient 1 (c.2393_2398del). This deletion (indicated by a red line below exon 18), if translated, results in loss of a methionine (M) and an alanine (A) residue (p.Met799_Ala800del) in the CatSper-epsilon protein. In addition to the 6 bp pathogenic deletion in exon 18, the position and genotype of six non-pathogenic intronic flanking single nucleotide polymorphisms (SNPs) are shown in the 22 exons of *CATSPERE*. The position of four non-pathogenic, highly variable, intronic SNPs and a 1 bp large homopolymeric 13/14 bp T tract in/del (rs10572994), are also indicated by red lines on the diagram.

**Figure 2:**
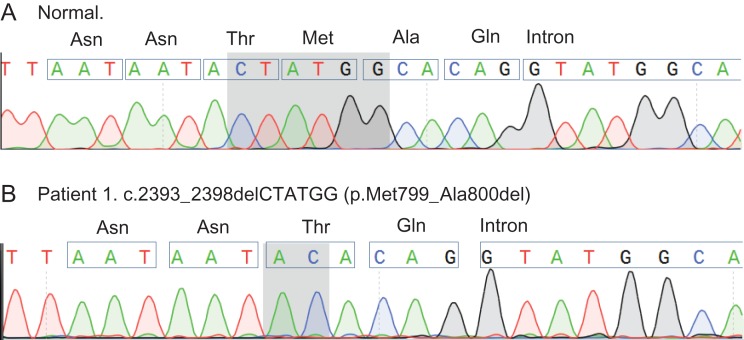
Sanger sequencing conformation of the initial exome sequencing results from patient 1. Highlighted is the position of the 6 bp CTATGG deletion (c.2393_2398del) in the normal trace (**A**) and the subsequent re-joining event, between the flanking adenine and cytosine bases shown in the trace for patient 1 (**B**). The normal sequence shows the position of Met 799 and Ala 800 amino acids that would be deleted if the variant protein is expressed in patient 1 (p.Met799_Ala800del).

Sequence variations, all of which have been previously described in the Ensembl genome browser database (GRCh37; http://grch37.ensembl.org), were identified in both *CATSPERE* (c1orf101) and *CATSPERZ* loci from patient 1 (Fig. 1). All intronic variations are predicted to be benign. However, patient 1 is homozygous for a highly deleterious (pathogenicity score of −11.3) in-frame 6-bp deletion in exon 18 (c.2393_2398delCTATGG, rs761237686) of *CATSPERE* which, if translated, would cause the loss of two amino acids in the extracellular domain (p.Met799_Ala800del) in isoform 1 of CatSper-epsilon (Fig. 2).

## Discussion

CatSper is a highly complex channel that consists of at least nine subunits and gene knock-out studies demonstrate that it is essential for male fertility in mouse (Ren *et al.*, 2001; Liu *et al.*, 2007; Qi *et al.*, 2007; Chung *et al.*, 2011, 2017). Identification of genetic abnormalities in *CATPSER1* and *CATSPER2* genes in subfertile men is consistent with similar importance of this channel in human spermatozoa. However, semen samples in these cases had multiple abnormalities, thus impaired fertility could not be conclusively attributed to the exclusive loss of CatSper (Avidan *et al.*, 2003; Smith *et al.*, 2013). In contrast, we reported a case of a stable lesion in CatSper function in sperm from patient 1 that failed to fertilize at IVF but had normal motility and concentration (Williams *et al.*, 2015). Genetic analysis revealed no significant changes in CatSper coding regions. However, in light of the recent identification of two new channel auxiliary subunits (CatSper-epsilon and CatSper-zeta; Chung *et al.*, 2017) we re-examined the genetics of this patient and now report the presence of an in-frame microdeletion in the putative extracellular coding region of *CATSPERE*. Our analysis shows a homozygous 6-bp frameshift deletion in exon 18 of *CATSPERE* (Figs 1 and 2).

CatSper-epsilon is predicted to be a single transmembrane spanning protein type II with a topology similar to CatSper-gamma and delta that localizes specifically to the plasma membrane in the same distinct quadrilateral arrangement shown for other subunits (Chung *et al.*, 2017) supporting the premise that it is an integral part of the mature CatSper signaling complex. Interestingly, the deleted amino acids are present within a pfam1502 conserved CatSper-delta superfamily domain. Alignment of corresponding extracellular sequences of human CatSper-epsilon and delta indicates they have a high-sequence homology (52% identical/similar amino acids), which may indicate a stabilizing function on channel assembly like that demonstrated for mouse CatSper-delta, which is critical for channel expression and male fertility (Chung *et al.*, 2011, Fig. 3).

**Figure 3:**
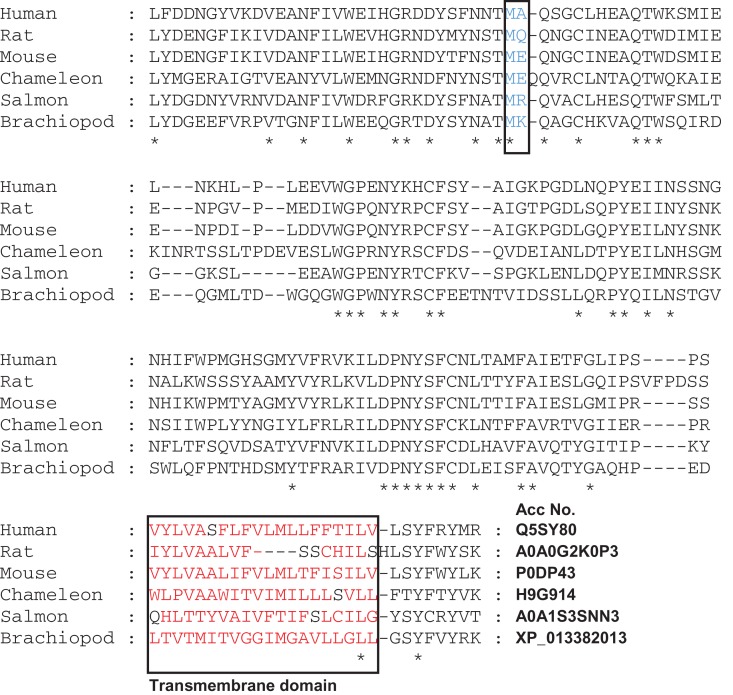
Alignment of a truncated region of CatSper-epsilon protein sequences (corresponding to 769aa–931aa of human CatSper-epsilon). Five selected evolutionary distant species were compared to the Human sequence using the EBI MUSCLE program. Stars (*) indicate conserved amino acids. The box with the blue lettering indicates the evolutionary conservation of the predicted deleted MA region in CatSper-epsilon of the patient 1 (p.Met799_Ala800del). The box containing red lettering illustrates a high density of hydrophobic amino acids that is the predicted transmembrane domain of the CatSper-epsilon orthologous proteins. The Uniprot or Genbank Accession numbers (Acc No.) for the different CatSper-epsilon proteins are given.

Determining the effect of the frameshift mutation on CatSper channel biogenesis during human spermatogenesis, as well as determining the stoichiometry of subunits and precise composition of the channel complex, is critical future work. Destablization of the channel complex may manifest through impaired CatSper-epsilon protein production due to transcript nonsense-mediated decay if the variant causes mis-splicing of exon 18. Alternatively, if the erroneous CatSper-epsilon transcript is translated, a highly conserved methionine (Fig. 3) and alanine will be absent in the putative extracellular domain of the protein, which is predicted to be highly deleterious to its conformation (PROVEAN pathogenicity score of −11.3) and potentially to channel assembly. As has been shown recently using super-resolution microscopy, the absence of even a single CatSper subunit can result in the ultimate destablization of the whole channel complex, and disorganize the precise nanodomain organization of the whole flagellum (Chung *et al.*, 2017). Our data add to a growing body of evidence to suggest an association between aberrations in CatSper genes and impaired fertility that may not manifest an overt phenotypical defect (Williams *et al.*, 2015) and therefore can cause unexplained infertility. Since heterologous expression of the functional CatSper channel complex cannot yet be achieved despite the decades of failed attempts by many research groups, the study of this channel is limited to its natural expression system—the mature spermatozoon. Therefore, the human genetics studies provide a valuable insight on the channel putative composition and the role of its subunits.

These initial observations show an association between a CatSper-epsilon variant and loss of CatSper channel function. Proof that this variant is the exclusive reason for the loss of channel function and fertilization competence will require further evidence. Generation of an equivalent *CATSPERE* mouse model is a conventional strategy and is potentially useful but a fundamental issue is that nothing is known about the molecular regulation of assembly or processing of human CatSper during spermatogenesis and its storage/transport through the epididymis, therefore species comparisons maybe flawed. *In vitro* recombinant studies to examine the expression and stability of the variant human protein have merit but only if functional expression is feasible (see above). An alternative approach is to use human genetic studies to investigate the channel putative composition and the role of its subunits. However, due to the low frequency of homozygous males in the population (~1 in 500 000 men. Ensembl/gnomAD) finding an identical case by screening, allowing replication of our study (Williams *et al.*, 2015), is unlikely. An effective strategy may require studies involving a multi-centre collaborative effort (Barratt *et al.*, 2017; 2018) to identify sentinel men through phenotypic screening (Kelly *et al.*, 2018) and/or clinical outcomes and perform genetic analysis and *in vitro* experiments (e.g. targeted quantitative proteomics and high-resolution imaging of CatSper *in situ*, Chung *et al.*, 2017).

In summary, we describe the first reported case of a man with a homozygous in-frame deletion in *CATSPERE* (r761237686) which may cause infertility through loss of mature CatSper channel function in spermatozoa. However, the precise molecular deficit remains to be elucidated and compounding genetic errors cannot be ruled out.
